# Integrated Information and Prospects for Gliding Mechanism of the Pathogenic Bacterium *Mycoplasma pneumoniae*

**DOI:** 10.3389/fmicb.2016.00960

**Published:** 2016-06-28

**Authors:** Makoto Miyata, Tasuku Hamaguchi

**Affiliations:** ^1^Department of Biology, Graduate School of Science, Osaka City UniversityOsaka, Japan; ^2^The OCU Advanced Research Institute for Natural Science and Technology, Osaka City UniversityOsaka, Japan

**Keywords:** cell architecture, evolution, electron microscopy, *Mollicute*, motility, sialylated oligosaccharide

## Abstract

*Mycoplasma pneumoniae* forms a membrane protrusion at a cell pole and is known to adhere to solid surfaces, including animal cells, and can glide on these surfaces with a speed up to 1 μm per second. Notably, gliding appears to be involved in the infectious process in addition to providing the bacteria with a means of escaping the host's immune systems. However, the genome of *M. pneumoniae* does not encode any of the known genes found in other bacterial motility systems or any conventional motor proteins that are responsible for eukaryotic motility. Thus, further analysis of the mechanism underlying *M. pneumoniae* gliding is warranted. The gliding machinery formed as the membrane protrusion can be divided into the surface and internal structures. On the surface, P1 adhesin, a 170 kDa transmembrane protein forms an adhesin complex with other two proteins. The internal structure features a terminal button, paired plates, and a bowl (wheel) complex. In total, the organelle is composed of more than 15 proteins. By integrating the currently available information by genetics, microscopy, and structural analyses, we have suggested a working model for the architecture of the organelle. Furthermore, in this article, we suggest and discuss a possible mechanism of gliding based on the structural model, in which the force generated around the bowl complex transmits through the paired plates, reaching the adhesin complex, resulting in the repeated catch of sialylated oligosaccharides on the host surface by the adhesin complex.

## Introduction

**Figure 1:**
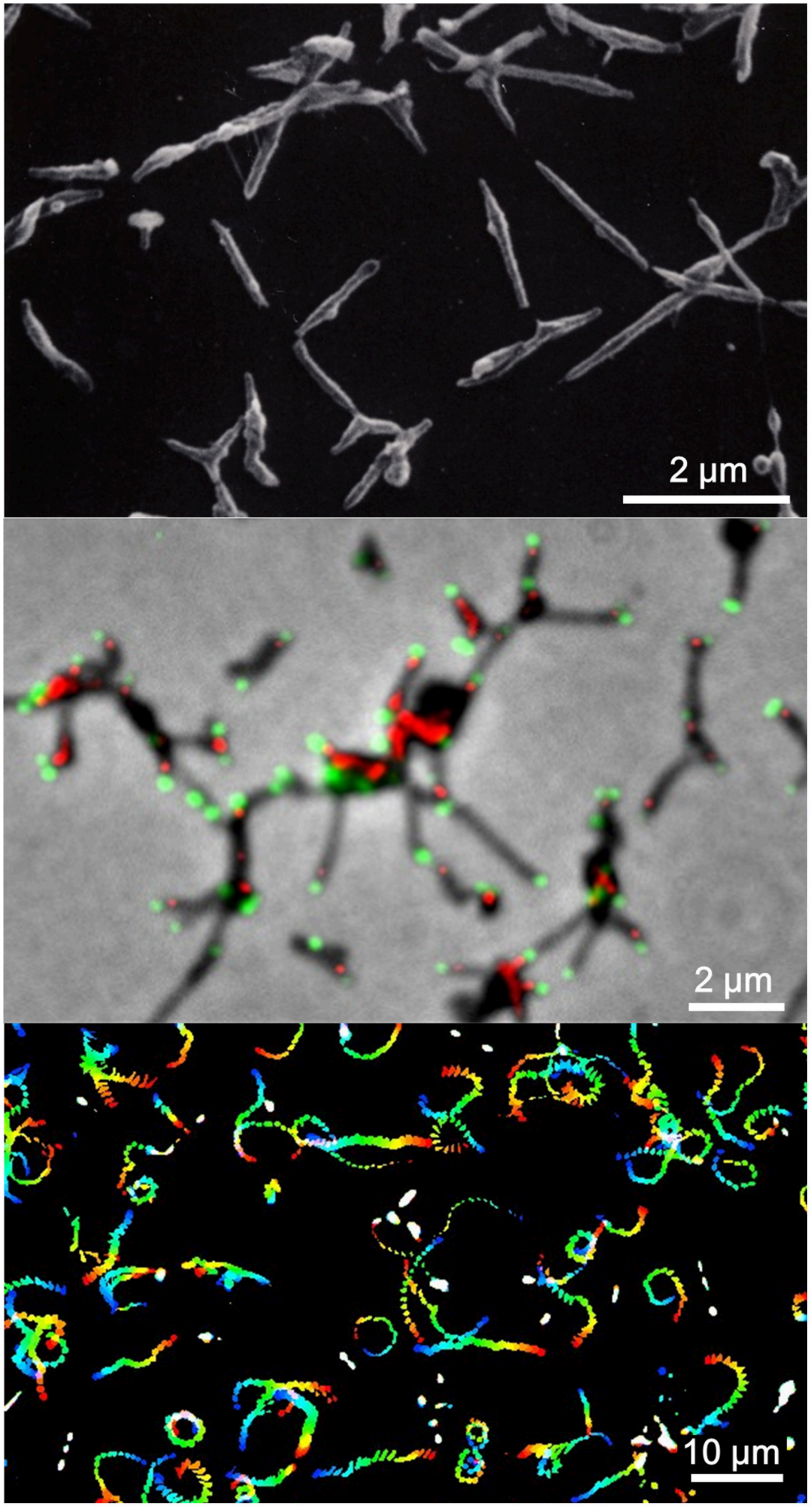
*****Mycoplasma pneumoniae*** cell structure and gliding. (Upper)** Scanning electron microscopy (SEM) of *M. pneumoniae*. **(Middle)** Merged image of phase contrast and fluorescence microscopy. Component proteins of the attachment organelle are marked in green and red. **(Lower)** Gliding track. The video frames are colored from red to blue with time and integrated for 20 s. The source video (Video 1) is available.

Mycoplasmas are parasitic and occasionally commensal bacteria that lack a peptidoglycan layer and have small genomes (Razin et al., 1998; Razin and Hayflick, 2010). *Mycoplasma pneumoniae* is a causative pathogen of human bronchitis and walking pneumonia as discussed in other reviews in this issue. This particular mycoplasma forms a membrane protrusion, called the “attachment organelle” or the “tip structure,” at a cell pole, binds to solid surfaces including those on the host, and glides in the direction of the protrusion (Figure 1; Video 1; Miyata, 2008; Miyata and Nakane, 2013; Balish, 2014). The gliding speed can reach up to 1 μm, one-half its cell length, per second (Radestock and Bredt, 1977; Kenri et al., 2004; Nakane and Miyata, 2009). This motility, combined with its cytadherence capacities, is involved in the *M. pneumoniae* infection process, enabling the cells to translocate from the tips of bronchial cilia to the host cell surface (Krunkosky et al., 2007; Prince et al., 2014). Interestingly, the genome sequence shows that this motility is not related to other known mechanisms of bacterial motility, nor does it involve motor proteins known to be involved in eukaryotic cell motility (Fraser et al., 1995; Dandekar et al., 2000; Jaffe et al., 2004b). In this article, we highlight what is currently understood concerning *M. pneumoniae* gliding and motility, providing insight into the function and mechanism of this process.

## Motility in *Mollicutes*

**Figure 2:**
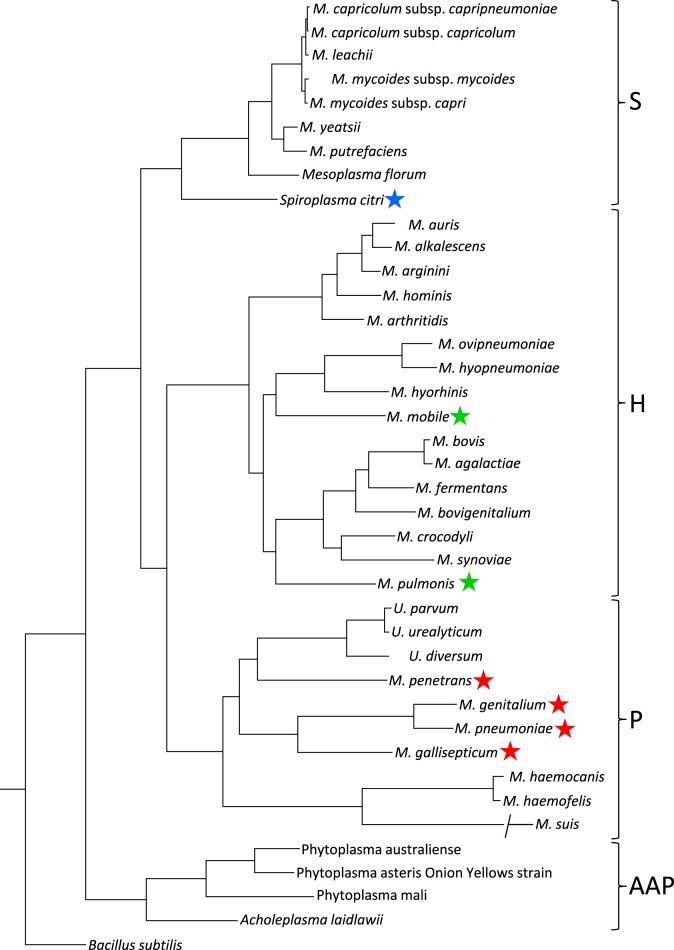
**Phylogenetic tree inferred using the multiple alignments of 79 proteins in conjunction with the maximum likelihood method**. Main phylogenetic subgroup are indicated: S, *Spiroplasma*; H, *Hominis*; P, *Pneumoniae*; AAP, *Acholeplasma/Anaeroplasma/Phytoplasma*. Motile species are marked by blue, green, and red stars for the motility types represented by *S. citri, M. mobile*, and *M. pneumoniae*, respectively. This figure was modified from study (Grosjean et al., 2014) through the courtesy of Dr Pascal Sirand-Pugnet.

The species in class *Mollicutes* represented by *Mycoplasmas* are classified into four subgroups (Figure 2; Grosjean et al., 2014). *Mycoplasma mobile* and *Mycoplasma pulmonis* in the *Hominis* subgroup and most species in the *Pneumoniae* subgroup have gliding capability. They share similar features in terms of the gliding process (i.e., they form a protrusion at a pole, bind to sialylated oligosaccharides fixed on solid surfaces through this protrusion, and glide in the direction of protrusion, at a speed ranging 0.1–4.5 μm per second). However, the proteins involved in the actual mechanisms underlying this motility in the two subgroups do not show any similarities in amino acid sequence or gene arrangement. These observations suggest that these two systems developed independently, achieving similar results through convergent evolution.

**Figure 3:**
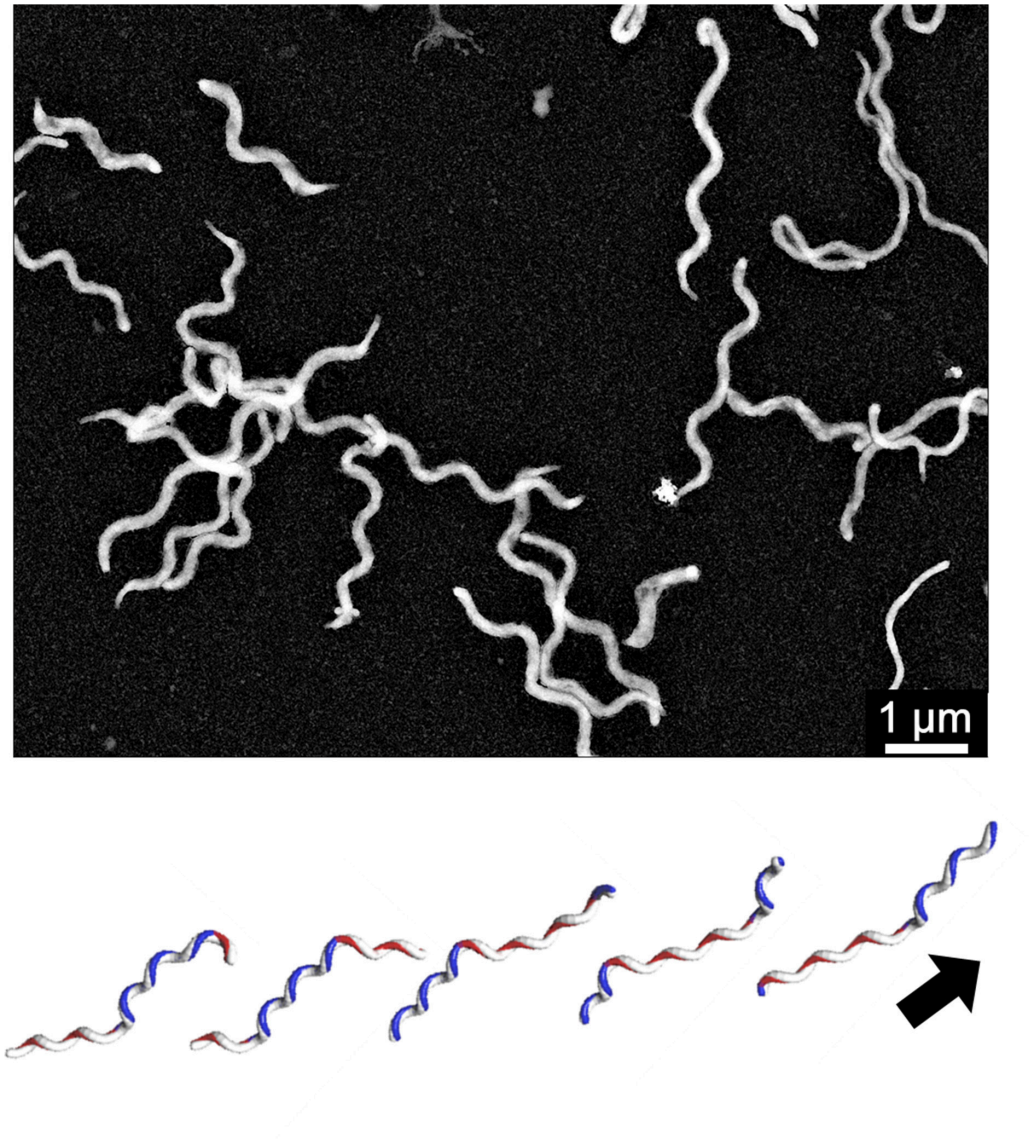
*****Spiroplasma*** swimming. (Upper)** Negative staining electron microscopy of *S. eriocheiris* cells. **(Lower)** Swimming scheme. A pair of kinks, or inversion points of helicity that travel from the front to the back, propel the cell in the direction of the arrow. This image is courtesy of Dr Hirofumi Wada.

The *Spiroplasma* subgroup contains the *Spiroplasma* and other species (Figure 2; Grosjean et al., 2014), all of *Spiroplasma* species swim in liquids of high viscosity (Shaevitz et al., 2005; Wada and Netz, 2009). Spiroplasmas propel viscous medium by propagating paired kinks formed at the front end to the back end along the helix axis (Figure 3), which is not related to spirochete swimming in which they rotate their flagella around the helix axis in the periplasmic space via the flagellar motors localized at both poles of the cell (Zhao et al., 2014). Notably, the spiroplasma genomes do not contain known genes related to other motility systems, with the exception of five homologs of MreB, known as “bacterial actin,” which functions as a component of the cytoskeleton in walled bacteria (Ku et al., 2014). Many species are known to have some type of motility, and all species in the *Pneumoniae, Hominis*, and *Spiroplasma* subgroups appear to utilize a common mechanism, although they may have developed autonomously.

## Development of three independent motility systems in *Mollicutes*

When studying the *Mollicute* species, one question that invariably arises is why and how did they develop these motility systems three separate times? Class *Mollicutes* developed from a low GC branch of Gram-positive bacteria i.e., phylum, *Firmicutes* including *Bacillus* and *Clostridium*, through their parasitic lifecycles (Weisburg et al., 1989). It also appears that the *Mollicutes* stopped synthesizing a peptidoglycan layer during their evolution, possibly as a way to evade the natural immune system of host animals, which often use Toll-like receptors to recognize peptidoglycans (Akira and Takeda, 2004). Changing the surface composition in this manner resulted in a subsequent change in cell shape, which became more flexible, as well as a reduction in genome size. Although bacterial flagella are well conserved in many bacterial species, and are effective for translocation through a wide variety of environments, including low and high viscosity liquids, and even liquid-solid interfaces (Kearns, 2010), these systems also require the mechanical support of a peptidoglycan layer. In fact, peptidoglycan binding sites on the flagella machinery are essential for rotation (Blair et al., 1991; De Mot and Vanderleyden, 1994; Koebnik, 1995). In *Mollicutes*, the evolutionary loss of bacterial flagella was again likely done in order to evade the host immune system, because flagellin is also a ligand of Toll-like receptors. However, motility is sometimes critical for parasitic bacteria to survive in host tissues, and in order to compensate for the lack of flagella, *Mollicutes* somehow developed new motility systems (Miyata, 2010; Miyata and Nakane, 2013; Balish, 2014; Miyata and Hamaguchi, 2016). The loss of the peptidoglycan layer may have actually help to mediate the development of this novel type of motility. In a cell, many housekeeping proteins are physically moving around the cell to perform various functions, including DNA replication, DNA repair, RNA synthesis, protein synthesis, protein degradation, ATP synthesis, transport, cell shape maintenance, cytokinesis, peptidoglycan synthesis, and so on. The development of a new motility system may have been achieved by transmitting these movements across the cell membrane to the scaffold outside. In walled bacteria, however, the movements inside cell cannot be transmitted outside, because the rigid peptidoglycan layer blocks the transmission of these movements. Perhaps, *Mollicutes* were capable of independently developing motility as many as three times, based on this advantage. For example, we recently suggested the possibility that *M. mobile* gliding may have originated from an accidental combination of F-type ATPase and adhesin movements (Miyata and Hamaguchi, 2016). Moreover, spiroplasma swimming may have originated from the cell shape maintenance system using MreB, a bacterial ortholog of eukaryotic actin (Kurner et al., 2005; Trachtenberg et al., 2008; Ozyamak et al., 2013). This kind of survival strategy adopted by *Mollicutes* may to some extent mimic that of the *Cephalopoda* species (Albertin et al., 2015), which over the course of their evolution quit making hard shells and instead developed flexible bodies with camouflaging abilities and different types of traveling systems (see other reviews in this issue).

## Purpose of motility

Although motile bacteria generally move to access nutrients and escape from waste and predators using “two-component systems” (Typas and Sourjik, 2015), neither type of mycoplasma gliding exhibits any obvious chemotaxis and no homologs of any two-component system genes have been identified in the mycoplasma genomes (Fraser et al., 1995; Dandekar et al., 2000; Jaffe et al., 2004b). However, we cannot rule out the possibility of chemotaxis in mycoplasmas (Kirchhoff et al., 1987), because the failure to detect chemotaxis may be a result of the experimental conditions used in these studies. Interestingly, the *Spiroplasma* species do show chemotaxis to amino acids, although they do not have any two-component system genes coded by their genomes (Daniels and Longland, 1984; Lo et al., 2013).

Even if the gliding mycoplasmas move randomly, such movements will enable them to reach conditions that are better for propagation, for example other spaces in the same and different animals. Gliding mycoplasmas repeatedly bind sialylated oligosaccharides on host cell surfaces to enable adhesion and gliding, as discussed below (Nagai and Miyata, 2006; Kasai et al., 2013, 2015). The binding affinity and gliding properties depend on structures that vary significantly in different tissues and in different animals. Mycoplasmas may detect the differences in the structure and reach the tissue covered with the sialylated oligosaccharide for which they have the highest affinity. Indeed, *M. mobile* stays longer on sialylated oligosaccharides with higher affinities than ones with lower affinities (Kasai et al., 2015). Prince et al. examined the infection efficiency of *M. pneumoniae* for a non-gliding mutant as well as other reduced gliding mutants with retained binding activity using a cell culture model of the human trachea (Prince et al., 2014). They found that the mutant cells could not establish robust infection like the unaltered *M. pneumoniae*, which efficiently moved from the tip of cilia to the host cell surface. Cytadherence of mycoplasmas, which is linked to gliding motility, has a well-established role in parasitism and pathogenicity (Razin and Jacobs, 1992; Razin et al., 1998). When a mycoplasma strain loses its capacity for cytadherence, it is easily removed by the host.

## The attachment organelle

**Figure 4:**
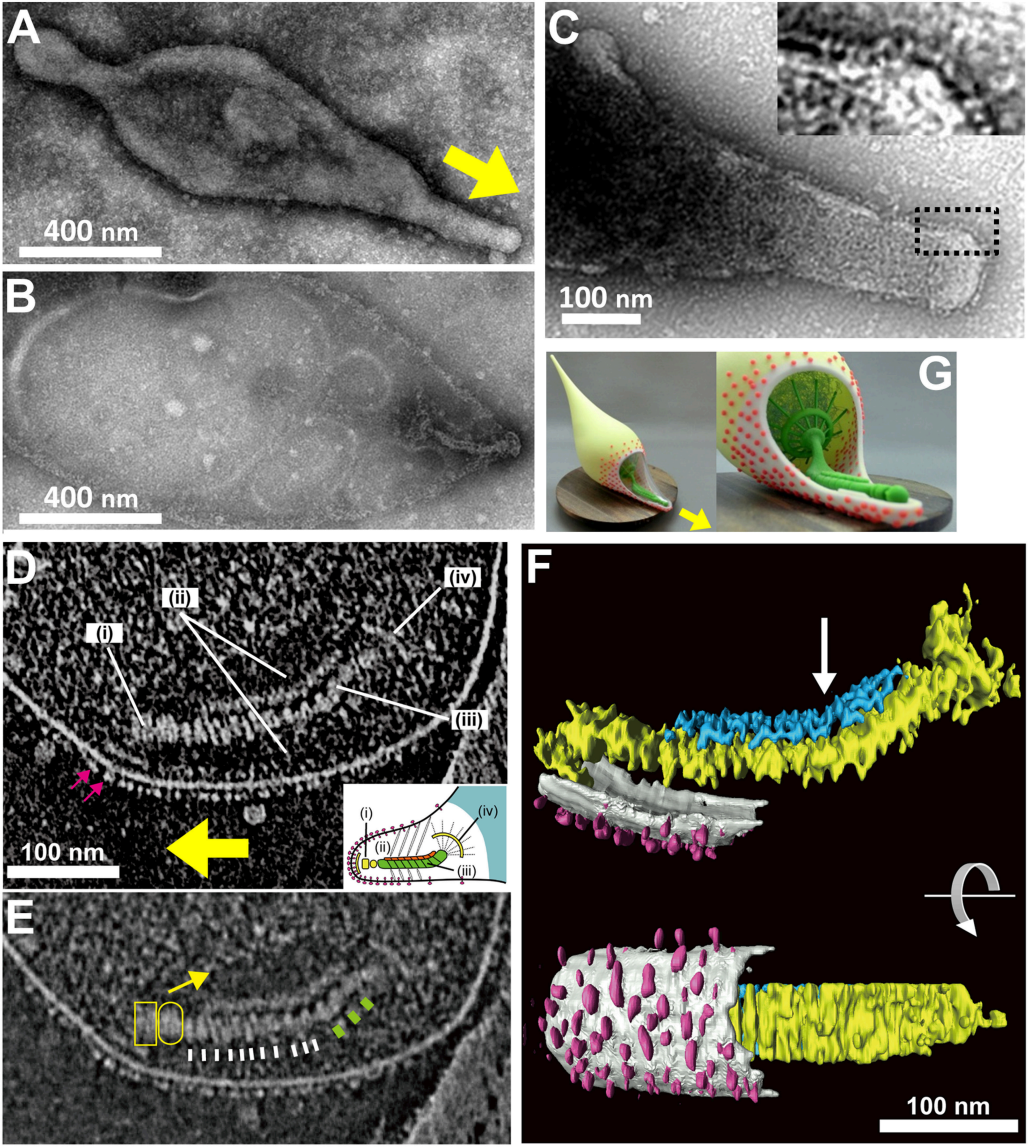
**Attachment organelle of ***Mycoplasma pneumoniae*** visualized by EM. (A)** Untreated cell bound to a carbon-coated grid. The membrane protrusion at the right pole is the attachment organelle. The gliding direction is shown by a yellow arrow. **(B)** Image of a cell treated with 1% Tween 20. The cell membrane was partially damaged, and the internal core remained at the right pole of the cell. **(A,B)** were modified from study (Nakane et al., 2015). **(C)** Nap structure. The area outlined by the dashed box is magnified in the inset. This panel was modified from study (Nakane et al., 2011). **(D)** ECT Image with a dense array of the knoblike particles on the membrane surface marked by pink arrows, and the terminal button (i), translucent area (ii), paired plates (iii), and bowl (wheel) complex (iv). The gliding direction is shown by a yellow arrow. Inset: schematic diagram of the attachment organelle. **(E)** Identical image to **(D)** but at lower contrast. The outline of the translucent area is marked with a yellow arrow. The thin and thick striations of the thick plate are marked by white and green bars, respectively. The terminal button is marked by a box and an oval corresponding to those in the inset schematic of **(D)**. **(F)** Rendered three-dimensional image of the tomogram shown in **(D)** depicting the knoblike particles (pink), membranes (light gray), and thin (blue) and thick (yellow) plates of the internal core. The bend is marked with an arrow. **(D–F)** were modified from study (Kawamoto et al., 2016). **(G)** Three dimensionally printed model of cell schematic. The surface and internal structures of the attachment organelle are colored red and green, respectively. The upper side of the cell membrane on the attachment organelle is omitted to show the internal structures. The *stl* format file to print out via 3D printer is available as a supplement. The gliding direction is shown by a yellow arrow.

*M. pneumoniae* basically has one attachment organelle per cell (Figures 1, 4; Bredt, 1968; Seto et al., 2001; Hasselbring et al., 2006; Nakane et al., 2015), suggesting that the formation of the nascent organelle occurs precisely coupling with cellular growth and division. This connection between attachment organelle formation and cell division has even been traced by fluorescence microscopy in both fixed and live cells (Seto et al., 2001; Hasselbring et al., 2006). These studies indicate that a nascent organelle is formed adjacent to the original one and moves laterally to the other pole. However, the cell images highlighting organelle formation vary in terms of the number of attachment organelles, with the next nascent organelle sometimes emerging before the end of cytokinesis.

It has been shown that the mycoplasma will bind to solid surfaces through the organelle and glide always in the direction of the organelle. In some genetic backgrounds the attachment organelle detaches from the cell body and glides away, suggesting that this organelle performs all of the activities essential for movement (Hasselbring and Krause, 2007a; García-Morales et al., 2016). The attachment organelle is approximately 300–350 nm long and the structures involved can be divided into those on the surface and those that are internal. The surface structures are composed of at least a few proteins that are directly involved in sialylated oligosaccharide binding on the host surfaces. The internal structures have several roles, including organelle formation and maintenance and force generation and transmission. The internal structures of the mycoplasma can be further divided into the translucent area and the core, which is composed of a terminal button, paired plates, and bowl complex (Miyata, 2008; Miyata and Nakane, 2013; Balish, 2014).

## Binding targets for gliding

**Figure 5:**
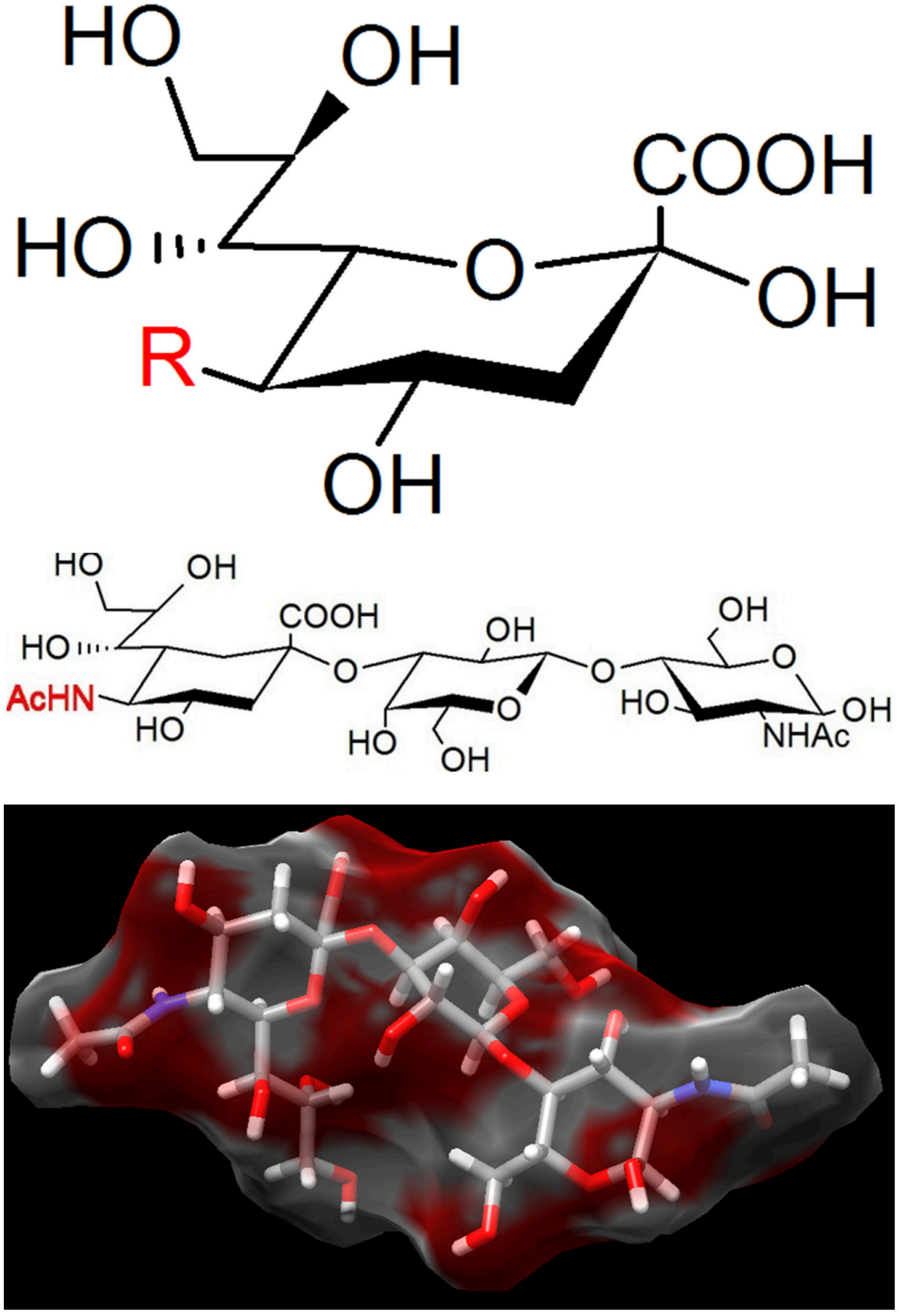
**The binding target of ***Mycoplasma pneumoniae***. (Upper)** Sialic acid is a generic form of the neuraminic acid derivatives, a monosaccharide with a nine-carbon backbone. Three representative structures are *N*-acetylneuraminic acid (R = AcNH), *N*-glycolylneuraminic acid (R = GcNH), and 2-keto-3-deoxynonic acid (KDN; R = OH). *N*-acetylneuraminic acid, the most common member, is occasionally called sialic acid. Sialic acids are found widely as the tip structure of oligosaccharide linked to glycoproteins and glycolipids on animal cell surfaces. **(Middle)** 3′-*N*-acetylneuraminyl-*N*-acetyllactosamine, recognized by *M. pneumoniae*. **(Lower)** The 3D structure of the same compound with **(Middle)** adapted from PubChem (CID: 14367963).

To identify the binding targets used during *M. pneumoniae* gliding, we focused on sialylated oligosaccharides (Figure 5) for two reasons: (i) these compounds have already been shown to be the binding targets of *M. mobile* gliding (Nagai and Miyata, 2006), and (ii) these compounds have been reported to be the binding target for static adherence of *M. pneumoniae* (Sobeslavsky et al., 1968; Manchee and Taylor-Robinson, 1969; Baseman et al., 1982a; Roberts et al., 1989). Our recent study showed that free molecules of sialylated oligosaccharides blocked the binding of *M. pneumoniae* to glass surface and also removed the gliding mycoplasma cells from the glass surfaces (Kasai et al., 2013). It is therefore probable that the gliding legs bind to the free 3′-*N*-acetylneuraminyl-*N*-acetyllactosamine when they are displaced from the binding target on the glass. We also analyzed the inhibitory effects of 16 chemically synthesized sialylated compounds on the gliding and binding of *M. pneumoniae* and concluded that the recognition of sialylated oligosaccharide by *M. pneumoniae* legs proceeds in a “lock-and-key” fashion, with the binding affinity being dependent on structural differences among the sialylated compounds examined. Further, this study also demonstrated that the binding of the leg and the sialylated oligosaccharide is cooperative, with Hill constants ranging from 1.5 to 2.3.

## P1 adhesin complex

**Figure 6:**
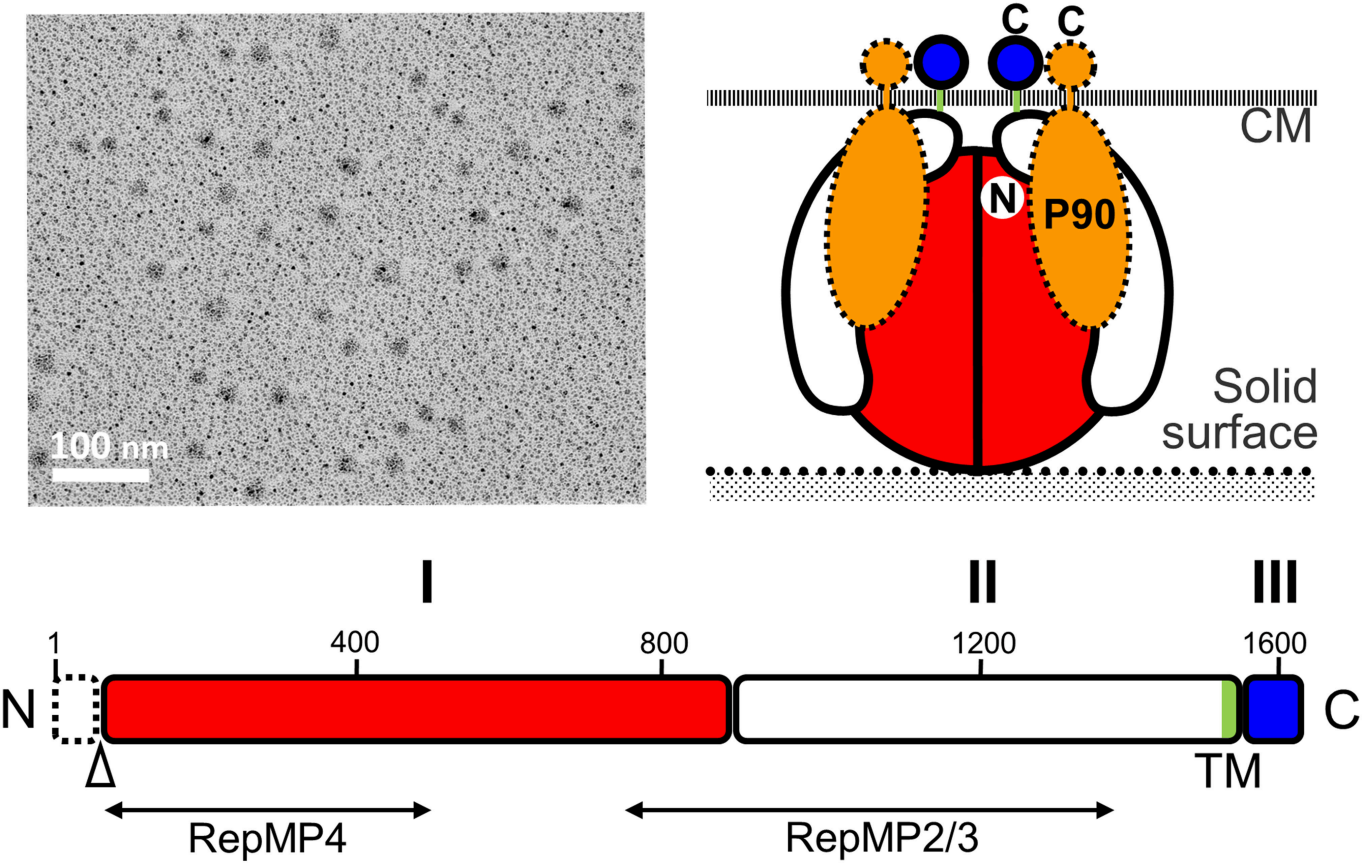
**Structure of the P1 adhesin complex. (Upper left)** Rotary-shadowing EM of the isolated P1 adhesin complex. **(Lower)** Schematic of the P1 adhesin amino acid sequence. The molecule is divided into domains I, II, and III, which are linked by predicted flexible hinges. Domain I is highly conserved. The transmembrane segment is colored green. The N-terminal 59 residues are removed during the maturation process at the position marked by an open triangle. The regions homologous to repetitive elements coded in different loci are indicated by lines marked RepMP4 and RepMP2/3. Domain III is inside the cell and probably interacts with other proteins in the cell. **(Upper right)** Schematic diagram of the P1 adhesin complex. Two molecules of P1 adhesin are predicted to fold into a globular complex with two molecules of P90. This figure was modified from study (Nakane et al., 2011) on permission.

The P1 adhesin (MPN141) is a 1627 amino acid protein that localizes over the whole surface of the attachment organelle (Seto et al., 2001; Seto and Miyata, 2003; Nakane et al., 2015) and is responsible for binding to solid surfaces (Baseman et al., 1982b; Feldner et al., 1982; Hu et al., 1982; Krause et al., 1982; Razin and Jacobs, 1992). Although P1 adhesin was identified as the adhesin involved in static binding, this protein also appears to function in the legs of gliding *M. pneumoniae* as monoclonal antibodies against P1 adhesin decrease gliding speed over time and eventually remove gliding cells from glass surfaces (Seto et al., 2005). Isolated P1 adhesin is cleaved at the C-terminal side of the 59th amino acid and forms a complex with a molecular mass of about 480 kDa that contains two molecules of P1 adhesin and two molecules of P90 (protein B, MPN142) (Figure 6; Hansen et al., 1979; Layh-Schmitt et al., 2000; Nakane et al., 2011). The complex forms a sphere approximately 20 nm in diameter when analyzed with rotary-shadowing electron microscopy (EM). P90 is encoded in tandem with P1 adhesin and is cleaved from another protein, P40 (protein C), after translation (Layh-Schmitt and Herrmann, 1992; Catrein et al., 2005). This cleavage is not seen in the ortholog of this protein in *Mycoplasma genitalium*, suggesting that P40 is also involved in the P1 adhesin complex on the organelle surface (Layh-Schmitt and Herrmann, 1994; Layh-Schmitt et al., 2000; Seto et al., 2001).

The P1 adhesin molecule can be divided into three domains (Nakane et al., 2011). Amino acid analysis indicates that domains I and III are well conserved between different species, and that there is a transmembrane region within domain II. It is thought that the binding site for sialylated oligosaccharides may be present in domain I because the amino acid sequences of the binding site would not be expected to evolve rapidly and domain III is predicted to be the internal part. However, the amino acid sequence does not show any clear similarities with any of the known sialylated oligosaccharide receptors, suggesting that P1 adhesin has a novel receptor structure.

The reduction of gliding speed by antibody binding to domain II may suggest that the actual movement of P1 adhesin is involved in the gliding mechanism (Seto et al., 2005). It is possible that the movements generated elsewhere may be transmitted to the P1 adhesin complex (Nakane et al., 2015), with the internal part, domain III, being responsible for force transmission from another internal part of the organelle. Alternatively, the distortion of the whole organelle shape or lateral array of the P1 adhesin complex may play this role (see below).

The sequence of P1 adhesin varies between clinical strains, resulting in structural changes in the immunodominant epitopes of the adhesin, which likely enable host immune system evasion (Nakane et al., 2011). The variation in the P1 adhesin sequence is thought to be generated by intragenomic recombination (Kenri et al., 1999; Spuesens et al., 2009).

## Surface structure

As *M. pneumoniae* binds to solid surfaces at the attachment organelle, surface structures responsible for binding would be expected to be located on this organelle. Nap structures, reminiscent of the raised surface of a cloth, can be observed at the surface of the membrane protrusion/attachment organelle using negatively stained EM (Figure 4; Baseman et al., 1982b; Hu et al., 1982). The nap structure on the surface likely corresponds to the P1 adhesin complex, because they localize on the organelle in a similar pattern, and the dimension and size of the P1 adhesin complex are comparable to those of the nap structures (Hu et al., 1982, 1987; Baseman et al., 1982b; Nakane et al., 2011). Notably, the nap structures cannot be clearly seen, because of the presence of the multiple layers. However, the surface structure of the attachment organelle could be reconstructed using electron cryotomography (ECT), in which the images of frozen specimens are captured at a series of angles relative to the electron beam, and the three dimensional structure is calculated. These structures appear to be “knob” shaped, with a length of 4–8 nm and a diameter of 8 nm (Henderson and Jensen, 2006; Seybert et al., 2006). Recent analysis by ECT suggested that the knob is identical to the nap in negative-staining EM, and showed that it forms a two-dimensional array of limited regularity on the surface (Kawamoto et al., 2016).

## Internal structure

**Figure 7:**
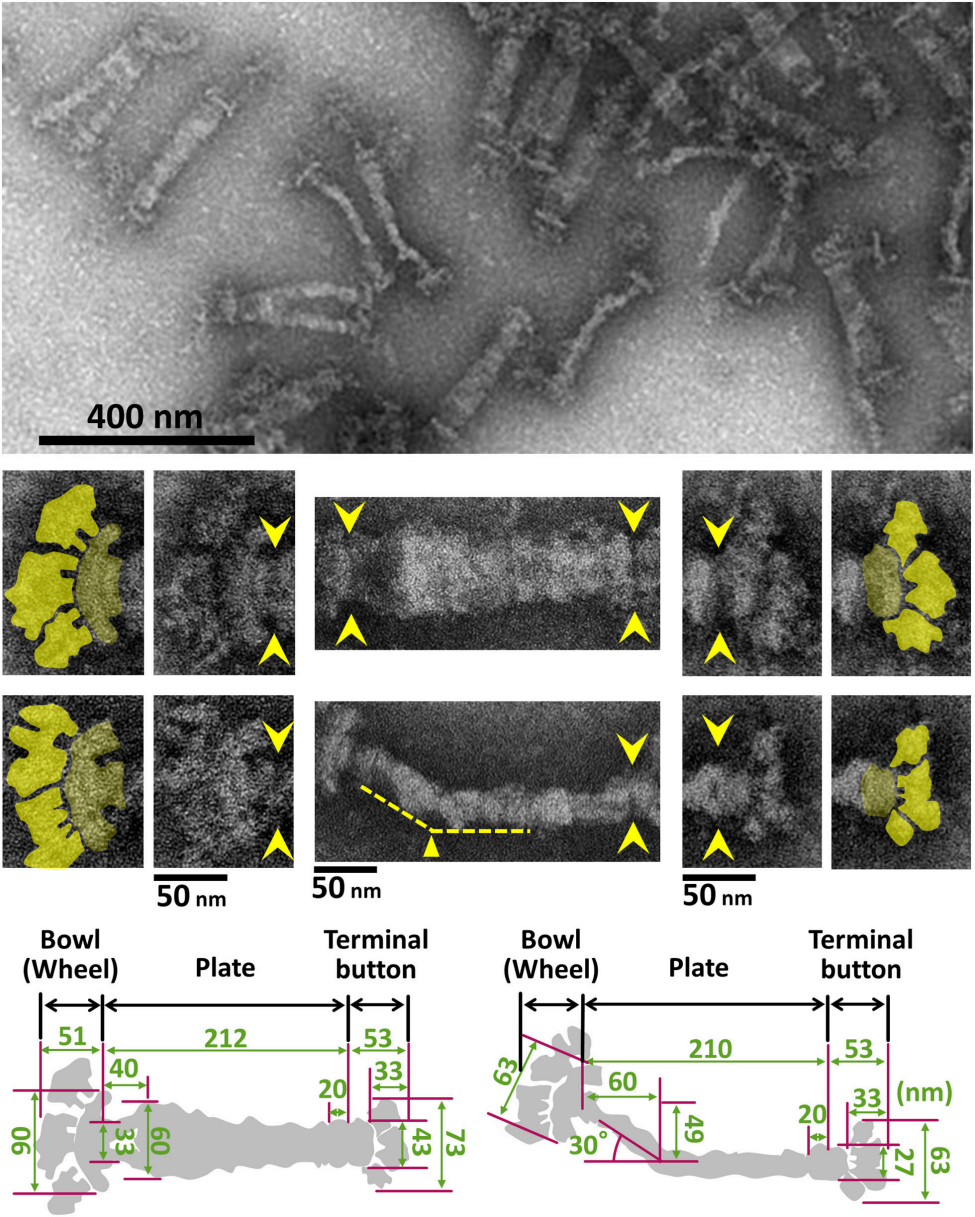
**Negative staining EM images of the isolated internal core of the attachment organelle**. **(Upper)** Core fraction. **(Middle)** Structural features of the internal core. Top and side views of the structures are shown in the upper and the lower panels, respectively. Yellow arrowheads indicate the boundaries between the core components. The original and colored images are shown in the adjacent panels for the bowl complex and terminal buttons in the left and the right, respectively. The thin plate is not visible in this preparation. A bend is observed in the side view around 60 nm from the back end, as marked by the yellow triangle. **(Lower)** Schematics and dimensions averaged for 40 structures. This figure was modified from study (Nakane et al., 2015).

The internal structure of the attachment organelle can be divided into two parts: the core structure and the translucent area. The core of *M. pneumoniae* was identified in the 1980s (Meng and Pfister, 1980; Gobel et al., 1981; Figure 4), and has also been referred to as the “electron dense core” in sectioning images (Wilson and Collier, 1976; Seto and Miyata, 2003), because it has a high electron density compared with other parts of the cell. The core can also be readily observed by EM in the center of the attachment organelle of the species in *Pneumoniae* subgroup after treating the cells with Triton X-100 (Meng and Pfister, 1980; Gobel et al., 1981; Hatchel and Balish, 2008; Nakane and Miyata, 2009; Relich et al., 2009). The core structure can be further divided into three parts: the “terminal button,” the “paired plates,” and the “bowl (wheel) complex” (Figure 7). These features are more evident in the cytoskeletal structure of *Mycoplasma gallisepticum*, which was previously described as an “asymmetrical dumbbell” (Nakane and Miyata, 2009). The details of this structure were further examined by ECT of whole cells (Hasselbring et al., 2006; Seybert et al., 2006; Kawamoto et al., 2016) and by negative staining EM of the core following isolation using centrifugation (Figure 7; Nakane et al., 2015).

## Component proteins of the attachment organelle

**Figure 8:**
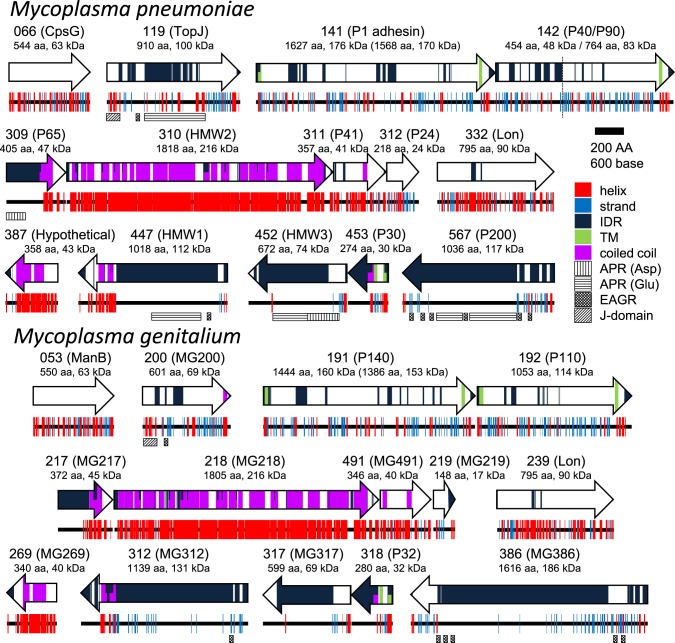
**Protein components of the attachment organelle**. Fifteen and fourteen proteins coded in nine loci on the genome are presented with their MPN and MG codes, respectively and the protein name. α-helix (helix), β-strand (strand), intrinsically disordered region (IDR), transmembrane segment (TM), coiled coil, Aspartic acid, and Proline rich region (APR Asp), Glutamic acid and Proline rich region (APR Glu), Enriched in Aromatic and Glycine Residues (EAGR), and the J-domain are predicted and marked. The amino acid number and predicted mass are shown beneath the protein name. The size after processing is shown in parentheses for MPN141 (P1 adhesin) and MG191 (P140). The cleavage site between P40 and P90 is shown by a broken line. *M. pneumoniae* and *M. genitalium* are presented as upper and lower panels, respectively.

**Figure 9:**
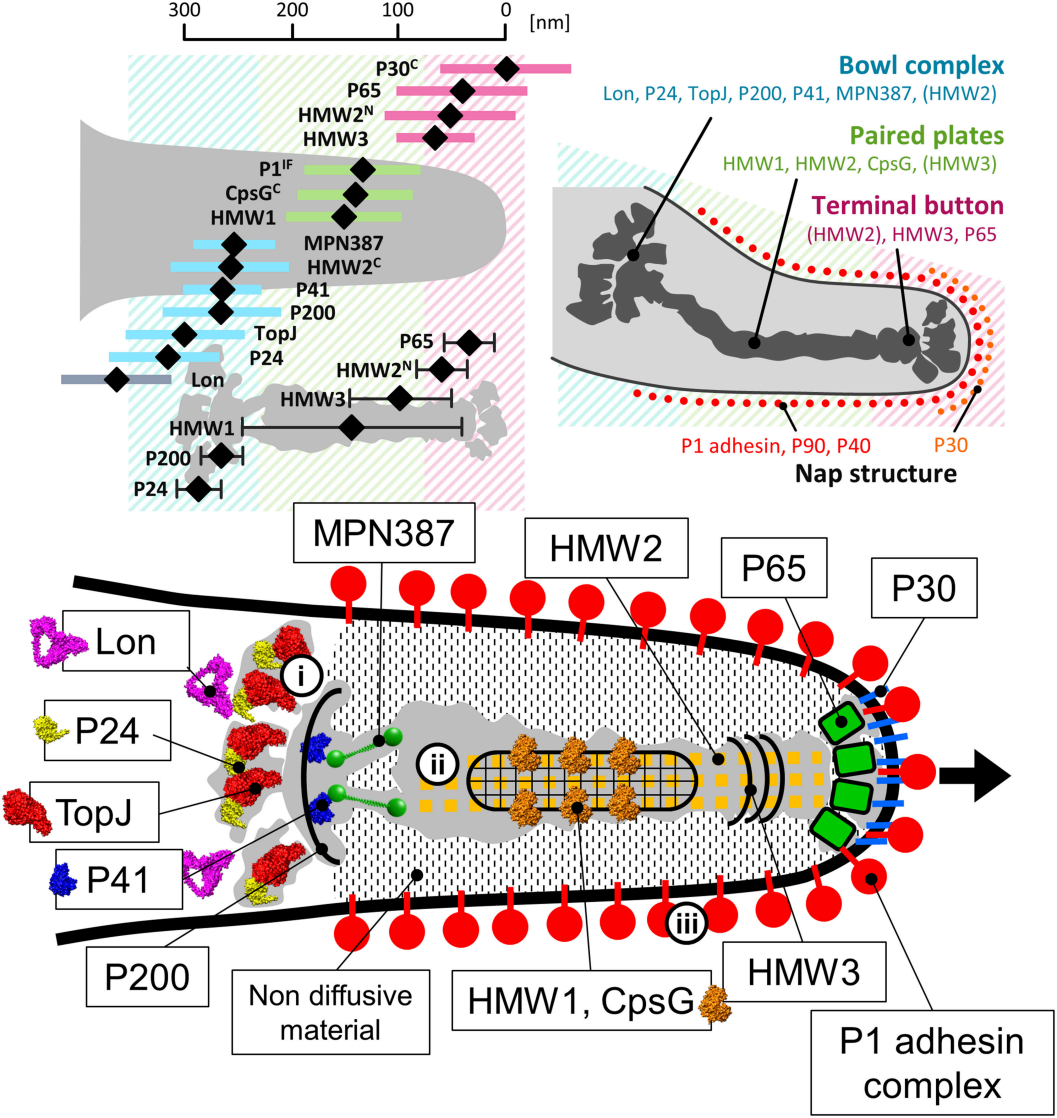
**Mapping and assignment of component proteins on the attachment organelle**. **(Upper left)** Protein positions based on fluorescence microscopy. The proteins were labeled by a monoclonal antibody for P1 adhesin, EYFP (Enhanced Yellow Fluorescent Protein) tagging at the C terminus if the protein name is marked “C,” or EYFP tagging at the N terminus for others. HMW2 was mapped at both the N- and C-terminus. The average position in individual cells and standard deviation relative to the cell edge are plotted with a black diamond and a colored bar, respectively. The mapping data based on immuno EM are plotted as a black diamond with a black bar in the lower. The proteins are mapped onto three localization groups, the terminal button (pink), the paired plates (green), and the bowl complex (blue). **(Upper right)** The attachment organelle including component proteins. Upper panels were modified from study (Nakane et al., 2015). **(Lower)** Assignment of the component proteins onto the schematic of the attachment organelle based on EM. The protein structures were predicted by Phyre2 (Kelley and Sternberg, 2009) whenever possible. In the gliding mechanism, the force may be generated at the bowl complex (i), transmitted through the paired plates (ii), and modulated by the P1 adhesin complex (iii). The gliding direction is shown by a black arrow.

To date, 15 proteins coded on 9 loci on the genome have been identified as components of the attachment organelle as shown in Figure 8 (Krause and Balish, 2004; Miyata and Nakane, 2013; Balish, 2014; Nakane et al., 2015), and each has recently been mapped on the organelle image systematically in nanometer order by fluorescence microscopy and immuno EM (Figure 9; Nakane et al., 2015). The features of this organization are summarized in Figure 8, including a new concept, intrinsically disordered region (IDR), which cannot form a stable three dimensional structure but can achieve it when it binds to other structures (Dyson and Wright, 2005). Interestingly, the sequences of P65 (MPN309), HMW1 (MPN447), HMW3 (MPN452), P30 (MPN453), and P200 (MPN567) are mostly predicted as IDR. We tried to assign all of 15 proteins onto the schematic of attachment organelle, based on the information that is currently available (Figure 9).

## P30, another surface protein

P30 (MPN453), which is comprised of 274 amino acid residues, has a transmembrane segment beginning from amino acid residue 72, with the carboxyl terminus oriented toward the outside of the cell (Figure 8; Chang et al., 2011). The subcellular localization of this protein is limited to the end of the attachment organelle, distinct from that of P1 adhesin (Figure 9; Seto et al., 2001; Seto and Miyata, 2003; Nakane et al., 2015). Notably, even in-frame deletions of 11 amino acid residues of this protein have been shown to mostly disrupt binding and gliding activities, although the stability of the protein and its localization are not significantly affected, showing that this protein functions as an important player in the gliding mechanism (Hasselbring et al., 2005; Chang et al., 2011). Moreover, chemical cross-linking studies suggest close proximity between P30 and P1 adhesin (Layh-Schmitt et al., 2000). The overexpression of the ortholog in *M. genitalium*, P32 (MG_318), rescues the instability of the P1 adhesin complex in mutant strains (García-Morales et al., 2016). The role of P32 is suspected to be similar to that of P30 in *M. pneumoniae* because P30 can be replaced by P32 in *M. pneumoniae* without significantly disrupting function (Relich and Balish, 2011). P65 (see below), a component of the internal structure likely interacts with P30, because depletion of either of them affects the other one's stability (Jordan et al., 2001; Chang et al., 2011; Hasselbring et al., 2012). These observations may suggest that P30 is linked to the front end of the internal structure, P65 and has a role in the proper alignment and movements of P1 adhesin complexes.

## Terminal button

A small piece of the membrane of Triton X-100 treated cells sometimes appears to be attached to the terminal button, suggesting a complex structure that includes the polar cell membrane. The terminal button can be divided into three major parts, the most distal of which is attached to the inner layer of the peripheral membrane proteins (Figure 7; Nakane et al., 2015). P65 (MPN309) and HMW3 (MPN452) have been mapped onto this structure (Stevens and Krause, 1992; Jordan et al., 2001; Seto et al., 2001; Seto and Miyata, 2003; Nakane et al., 2015). Considering the close spatial and functional relations between P30 and P65, as well as the positioning of the component proteins, P65 likely interacts with the internal domain of P30 (Figure 9; Jordan et al., 2001; Chang et al., 2011; Hasselbring et al., 2012). Further, the tight binding of the terminal button to the front side of the membrane may be achieved through this interaction. P30 may fix dozens of P1 adhesin complexes at the front end to form the initial core of the P1 adhesin complex array covering the organelle through the lateral interactions among the complexes. P65, on the other hand, may determine the gliding direction by modifying the angle of the organelle relative to the cell axis as deletion of MG217 in *M. genitalium*, the ortholog of P65, changes the gliding direction, consistent with this function (Burgos et al., 2008). The N- and C-terminus of HMW2 (MPN310), a coiled-coil protein were also mapped to the terminal button and bowl complex, respectively (Nakane et al., 2015). Probably, HMW3 bundles the array of HMW2 at the back side of terminal button.

## Paired plates

The paired plates are composed of paired striated plates separated by a gap of about 7 nm (Hasselbring et al., 2006; Henderson and Jensen, 2006; Seybert et al., 2006; Kawamoto et al., 2016). The paired plates appear flexible and bend approximately 30 degrees just proximal to their middle (Figure 7). This bend suggests that *M. pneumoniae* cells have three axes, front-back, upper-lower and left-right. The actual alignment of these axes relative to the solid surface during gliding is unknown. The localization of the N- and C-terminus at the terminal button and bowl complex, respectively, shows that HMW2 protein forms a dimer and a parallel bundle (Figures 8, 9; Krause et al., 1997; Nakane et al., 2015). In the thick plate, striations may correspond to eleven coiled-coil regions intermittently appearing in the sequence of the 1818 amino acids present in HMW2 (Dandekar et al., 2000; Letunic et al., 2015; Kawamoto et al., 2016). The thin plate featured with a stable hexagonal lattice (Kawamoto et al., 2016) is likely composed of HMW1 (MPN447) and CpsG (MPN066).

The paired plates likely function as the scaffold for formation of the attachment organelle, as both of HMW1 (MPN447) and HMW2 (MPN310) are essential for the early stage of organelle formation (Popham et al., 1997; Hahn et al., 1998; Seto and Miyata, 2003; Kenri et al., 2004; Burgos et al., 2007, 2008; Bose et al., 2009). The paired plates are also expected to have a critical role in gliding. Indeed, an *M. genitalium* mutant can glide without MG218, the ortholog of HMW2, if P32 (MG318) the ortholog of P30 (MPN453) was overexpressed, however gliding speed was 100-fold decreased (García-Morales et al., 2016).

## Bowl complex

Based on their examination of EM cryosections, Hegermann et al. have suggested that the striated paired plate is attached at the proximal end to a “wheel (bowl) complex” with fibrils, which connects the complex to the cell body (Hegermann et al., 2002; Mayer, 2006). A similar structure, called the “bowl,” has been found at the position of the wheel complex in *M. pneumoniae* by ECT, although fibrils were not observed (Henderson and Jensen, 2006; Seybert et al., 2006; Kawamoto et al., 2016). In contrast, fibrils were observed in negatively stained EM images of the “asymmetrical dumbbell” isolated from *M. gallisepticum* cells, suggesting that fibrils are probably present in the core in *M. pneumoniae* (Nakane and Miyata, 2009). In the reduced contrast ECT images, the identification of thin fibers is sometimes more difficult than in other methods. MPN387, the C terminus of HMW2 (MPN310), P41 (MPN311), P200 (MPN567), TopJ (MPN119), P24 (MPN312), and Lon (MPN332) appear to be localized on the bowl complex (Kenri et al., 2004; Jordan et al., 2007; Cloward and Krause, 2009; Nakane et al., 2015; Figure 9). The bowl complex likely has a role in connecting the attachment organelle to other parts of the cell, because in mutant strains of *M. pneumoniae* and *M. genitalium* that lack P41 or MG491, respectively, the attachment organelle occasionally detaches from the cell body and glides independently (Hasselbring and Krause, 2007a,b; García-Morales et al., 2016). The bowl complex may also be responsible for the generation or transmission of force, because mutation of the P200 or TopJ orthologs in *M. genitalium* (MG386 and MG200, respectively) results in mutants that can adhere, but have less gliding capacity, common with the character of P200 mutant of *M. pneumoniae* (Pich et al., 2006; Jordan et al., 2007). Moreover, the mutants depleted for MPN387 cannot glide, although they also retain their cytadherence properties (Hasselbring et al., 2005). The mapped position (Nakane et al., 2015) and the predicted molecular shape of the MPN387 protein suggested that it fits onto the front side of bowl complex. Thus, MPN387 may bridge the bowl complex to the back side of paired plates to transmit force. We have also predicted the molecular shapes of P41, TopJ, P24, and Lon, based on their amino acid sequences (Kelley and Sternberg, 2009), and have assign those images onto the core image based on recent mapping results (Figure 9).

## Translucent area

The core is surrounded by an electron lucent area from which the dense complexes are excluded (Wilson and Collier, 1976; Shimizu and Miyata, 2002; Seto and Miyata, 2003). This area is unlikely an artifact of chemical fixation and dehydration because it is also observed following rapid freezing (Henderson and Jensen, 2006; Seybert et al., 2006; Kawamoto et al., 2016). Hegermann et al. examined the structure of this area by treating fixed cells with Triton X-100 and suggest that thin filamentous structures connect the electron-dense core and the periphery of the cell (Hegermann et al., 2002). Henderson and Jensen also proposed that the translucent area was created by the exclusion of macromolecules via the repeated movement of the electron-dense core (Henderson and Jensen, 2006). However, this hypothesis is unlikely because the translucent area can be observed in all cells even if they are not in conditions optimized for gliding (Shimizu and Miyata, 2002; Seto and Miyata, 2003). This area may be occupied by stiff, less diffusive materials invisible in EM, which may play a role in transmitting the movements of the paired plates originated in the bowl complex (Figure 9; Henderson and Jensen, 2006; Seybert et al., 2006; Kawamoto et al., 2016). This invisible material is unlikely domain III of P1 adhesin, because the translucent area can be observed in a mutant lacking the P1 adhesin complex (Seto and Miyata, 2003; Kawamoto et al., 2016).

## Formation and origin of the attachment organelle

The gene arrangements of many of the component proteins in the genome agree with their suspected protein alignments in the attachment organelle (Figures 8, 9). The gene order, MPN309 (P65) to MPN312 (P24), agrees with the protein order in the core from the front end to the back end. Another gene order MPN453 (P30) to MPN452 (HMW3) also agrees with the protein order. Notably, the proteins of P1 adhesin complex are coded tandemly as MPN141 (P1 adhesin) and MPN142 (P40/P90) and are likely synthesized continuously and assembled into the complexes (Inamine et al., 1988; Krause et al., 1997; Waldo et al., 1999). This assumption is consistent with observations that 5.5% of internal cores have a shape that is branched at the front side (Nakane and Miyata, 2009; Nakane et al., 2015). The proteins featured the most with IDR [i.e., TopJ (MPN119), P65 (MPN309), HMW1 (MPN447), HMW3 (MPN452), P30 (MPN453), and P200 (MPN567)] may be assembled into the existing structures immediately after they are synthesized. The gene clustering of these proteins may also suggest the evolution stages. For example, in the early stage, the sialic acid receptor of the P1 adhesin complex was supported mechanically by the core composed of P65, HMW2, P41, and P24. In the next stage, the combination of the adhesin and the core was stabilized by P30 and HMW3. In the final stage, other proteins, including a force generator, were integrated into the system. Interestingly, we can find one ATPase, Lon (MPN332), included in the 15 component proteins, and while it has high sequence similarity with Lon proteases, represented by 46% identity with that from *Clostridium spiroforme* (WP_050752792.1), it is possible that this protein functions in the attachment organelle.

## Energy source

Information about the energy source is indispensable in clarifying the mechanisms involved in any kinds of motility. Most of bacterial motility systems are based on membrane potential rather than ATP, including bacterial flagella, *Myxococcus* gliding, *Flavobacterium* gliding, and so on, with only pili based motility utilizing ATP energy (Jarrell and McBride, 2008). Probably, this is because the majority of actively moving bacteria at first pool their energy to use, as a membrane potential through respiration. In *M. mobile*, on the contrary, the energy of motility is provided by ATP (Jaffe et al., 2004a; Uenoyama and Miyata, 2005; Kinosita et al., 2014), consistent with the above assumption, because generally *Mollicutes* pool their energy as high energy phosphate compounds, like ATP (Razin et al., 1998). *M. mobile* has been proven to be driven by the energy of ATP through the use of cellular “ghosts” that have damaged membranes, which could be reactivated by the addition of ATP (Uenoyama and Miyata, 2005; Kinosita et al., 2014). We have tried similar experiments with *M. pneumoniae*, but were unable to reactivate the motility of the “ghosts.” However, this does not promptly suggest that *M. pneumoniae* is driven by a different energy source. All the essential parts of the gliding machinery are required after the damage is inflicted on the cell envelope by the detergent, so the failure of these experiments may reflect the sensitivity of the gliding mechanism of *M. pneumoniae* to the detergent. The deletion of Ser/Thr protein kinase gene (prkC; MPN248) or its cognate phosphatase gene (prpC; MPN247) also influences the frequency and speed of gliding as well as the phosphorylation levels of the component proteins in the attachment organelle (Dirksen et al., 1994; Schmidl et al., 2010; Page and Krause, 2013), suggesting that changing the local charge coupled with phosphorylation is involved in the gliding mechanism.

These observations, however, do not deny the possibility that membrane potential could be the direct energy source as mycoplasmas also possess membrane potential like other bacterial species, which is likely caused by the F-type ATPase on the membrane using ATP energy (Benyoucef et al., 1981).

## Suggestion for gliding mechanism

We have been studying the gliding mechanism of *M. mobile* since 1997, and have suggested a “centipede model” mechanism in which *M. mobile* repeatedly catches, pulls, and releases sialylated oligosaccharides on host surfaces, based on the movements generated by an ATPase that are transmitted through the cell membrane (Miyata, 2008, 2010; Miyata and Nakane, 2013; Miyata and Hamaguchi, 2016). Unfortunately, only some common features appear to exist between the gliding mechanisms of *M. mobile* and *M. pneumoniae* (Miyata, 2008; Miyata and Nakane, 2013).

**Figure 10:**
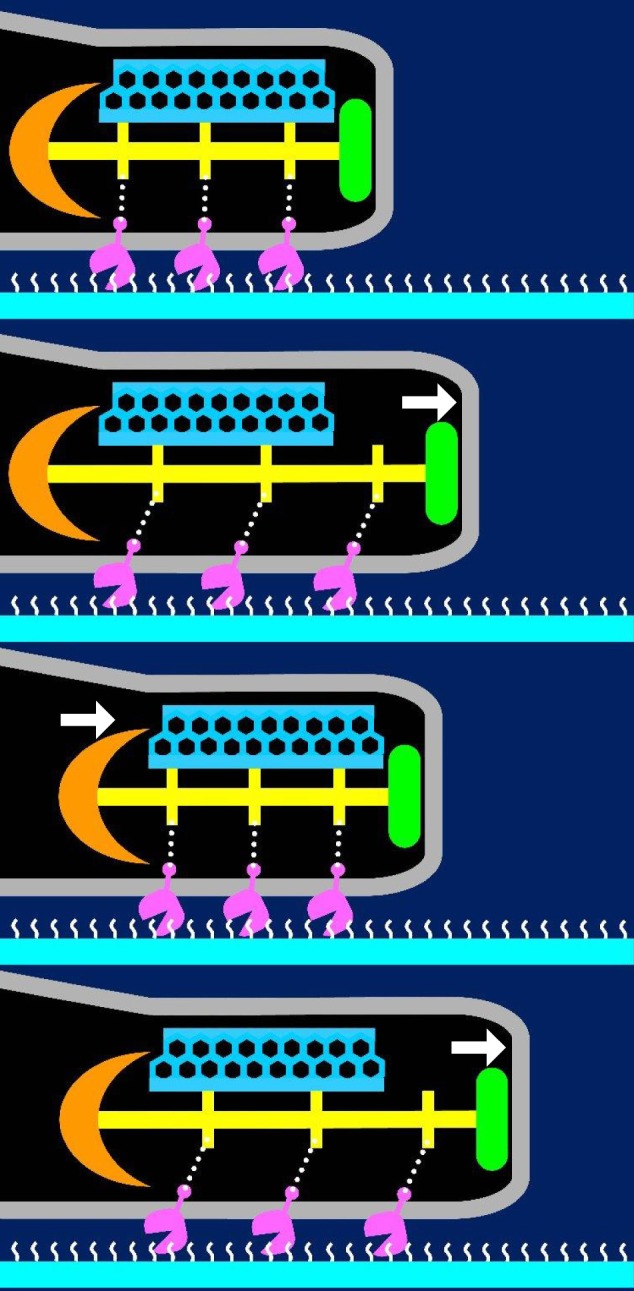
**Suggested mechanism underlying gliding**. The P1 adhesin, colored pink is responsible for binding to sialylated oligosaccharides on the solid surface colored white. The thin and thick plates, colored blue and yellow, respectively, featured a rigid hexagonal lattice and a striation with a highly variable pitch. The terminal button is colored green. The compression and extension of the thick plate may cause shortening and elongation of the attachment organelle, respectively. The thick plate changes its pitch and induces directed detachment of P1 adhesin from SOs, and leading to cell displacement from left to right. This figure was modified from study (Kawamoto et al., 2016).

Here, we have suggested a possible model for the gliding mechanism utilized by *M. pneumoniae* that integrates all of the known information (Figures 9, 10; Nakane et al., 2015; Kawamoto et al., 2016). Movements for gliding may be generated in the bowl complex and transmitted efficiently to the paired plates fixed to the cell front through P65 and P30 in the terminal button. Then, extension and retraction of the attachment organelle will occur. This movement is transmitted to the P1 adhesin complexes through distortion of the translucent area and/or the complex array on the surface. The P1 adhesin complexes will then repeat a catch-pull-release cycle with sialylated oligosaccharides on the host surface. The attachment organelle will then pull the other parts of the cell connected to the back end of the bowl complex, resulting in cellular movement. Additional work is warranted to further elucidate the proteins and pathways involved in this process.

## Author contributions

TH analyzed protein and sugar structures and prepared Figures 5, 8 and a part of 9. MM prepared other figures and text. Both checked the completed version.

## Funding

This work was supported by a Grant-in-Aid for Scientific Research on the Innovative Area “Harmonized Supramolecular Motility Machinery and Its Diversity” (MEXT KAKENHI Grant Number 24117002), and by a Grant-in-Aid for Scientific Research (B) (MEXT KAKENHI Grant Number 24390107) to MM.

### Conflict of interest statement

The authors declare that the research was conducted in the absence of any commercial or financial relationships that could be construed as a potential conflict of interest.

## References

[B1] Akira S. Takeda K. (2004). Toll-like receptor signalling. Nat. Rev. Immunol. 4, 499–511. 10.1038/nri1391 15229469

[B2] Albertin C. B. Simakov O. Mitros T. Wang Z. Y. Pungor J. R. Edsinger-Gonzales E.. (2015). The octopus genome and the evolution of cephalopod neural and morphological novelties. Nature 524, 220–224. 10.1038/nature14668 26268193 PMC4795812

[B3] Balish M. F. (2014). *Mycoplasma pneumoniae*, an underutilized model for bacterial cell biology. J. Bacteriol. 196, 3675–3682. 10.1128/JB.01865-14 25157081 PMC4248795

[B4] Baseman J. B. Banai M. Kahane I. (1982a). Sialic acid residues mediate *Mycoplasma pneumoniae* attachment to human and sheep erythrocytes. Infect. Immun. 38, 389–391. 6815092 10.1128/iai.38.1.389-391.1982 PMC347745

[B5] Baseman J. B. Cole R. M. Krause D. C. Leith D. K. (1982b). Molecular basis for cytadsorption of *Mycoplasma pneumoniae*. J. Bacteriol. 151, 1514–1522. 6809731 10.1128/jb.151.3.1514-1522.1982 PMC220433

[B6] Benyoucef M. Rigaud J. L. Leblanc G. (1981). The electrochemical proton gradient in *Mycoplasma* cells. Eur. J. Biochem. 113, 491–498. 10.1111/j.1432-1033.1981.tb05090.x 6260481

[B7] Blair D. F. Kim D. Y. Berg H. C. (1991). Mutant MotB proteins in *Escherichia coli*. J. Bacteriol. 173, 4049–4055. 2061285 10.1128/jb.173.13.4049-4055.1991 PMC208053

[B8] Bose S. R. Balish M. F. Krause D. C. (2009). *Mycoplasma pneumoniae* cytoskeletal protein HMW2 and the architecture of the terminal organelle. J. Bacteriol. 191, 6741–6748. 10.1128/JB.01486-08 19717588 PMC2795293

[B9] Bredt W. (1968). Motility and multiplication of *Mycoplasma pneumoniae*. A phase contrast study. Pathol. Microbiol. 32, 321–326. 4892712 10.1159/000162074

[B10] Burgos R. Pich O. Q. Querol E. Piñol J. (2007). Functional analysis of the *Mycoplasma genitalium* MG312 protein reveals a specific requirement of the MG312 N-terminal domain for gliding motility. J. Bacteriol. 189, 7014–7023. 10.1128/JB.00975-07 17675381 PMC2045215

[B11] Burgos R. Pich O. Q. Querol E. Piñol J. (2008). Deletion of the *Mycoplasma genitalium* MG_217 gene modifies cell gliding behaviour by altering terminal organelle curvature. Mol. Microbiol. 69, 1029–1040. 10.1111/j.1365-2958.2008.06343.x 18573175

[B12] Catrein I. Herrmann R. Bosserhoff A. Ruppert T. (2005). Experimental proof for a signal peptidase I like activity in *Mycoplasma pneumoniae*, but absence of a gene encoding a conserved bacterial type I SPase. FEBS J. 272, 2892–2900. 10.1111/j.1742-4658.2005.04710.x 15943820

[B13] Chang H. Y. Jordan J. L. Krause D. C. (2011). Domain analysis of protein P30 in *Mycoplasma pneumoniae* cytadherence and gliding motility. J. Bacteriol. 193, 1726–1733. 10.1128/JB.01228-10 21257768 PMC3067664

[B14] Cloward J. M. Krause D. C. (2009). *Mycoplasma pneumoniae* J-domain protein required for terminal organelle function. Mol. Microbiol. 71, 1296–1307. 10.1111/j.1365-2958.2009.06602.x 19183275 PMC5833977

[B15] Dandekar T. Huynen M. Regula J. T. Ueberle B. Zimmermann C. U. Andrade M. A.. (2000). Re-annotating the *Mycoplasma pneumoniae* genome sequence: adding value, function and reading frames. Nucleic Acids Res. 28, 3278–3288. 10.1093/nar/28.17.3278 10954595 PMC110705

[B16] Daniels M. Longland J. M. (1984). Chemotactic behavior of *Spiroplasmas*. Curr. Microbiol. 10, 191–194. 10.1007/BF01627253

[B17] De Mot R. Vanderleyden J. (1994). The *C*-terminal sequence conservation between OmpA-related outer membrane proteins and MotB suggests a common function in both Gram-positive and Gram-negative bacteria, possibly in the interaction of these domains with peptidoglycan. Mol. Microbiol. 12, 333–334. 10.1111/j.1365-2958.1994.tb01021.x 8057857

[B18] Dirksen L. B. Krebes K. A. Krause D. C. (1994). Phosphorylation of cytadherence-accessory proteins in *Mycoplasma pneumoniae*. J. Bacteriol. 176, 7499–7505. 8002573 10.1128/jb.176.24.7499-7505.1994 PMC197206

[B19] Dyson H. J. Wright P. E. (2005). Intrinsically unstructured proteins and their functions. Nat. Rev. Mol. Cell Biol. 6, 197–208. 10.1038/nrm1589 15738986

[B20] Feldner J. Gobel U. Bredt W. (1982). *Mycoplasma pneumoniae* adhesin localized to tip structure by monoclonal antibody. Nature 298, 765–767. 10.1038/298765a0 7110314

[B21] Fraser C. M. Gocayne J. D. White O. Adams M. D. Clayton R. A. Fleischmann R. D.. (1995). The minimal gene complement of *Mycoplasma genitalium*. Science 270, 397–403. 10.1126/science.270.5235.397 7569993

[B22] García-Morales L. González-González L. Querol E. Piñol J. (2016). A minimized motile machinery for *Mycoplasma genitalium*. Mol. Microbiol. 100, 125–138. 10.1111/mmi.13305 26712501

[B23] Göbel U. Speth V. Bredt W. (1981). Filamentous structures in adherent *Mycoplasma pneumoniae* cells treated with nonionic detergents. J. Cell Biol. 91, 537–543. 10.1083/jcb.91.2.537 6796593 PMC2111982

[B24] Grosjean H. Breton M. Sirand-Pugnet P. Tardy F. Thiaucourt F. Citti C.. (2014). Predicting the minimal translation apparatus: lessons from the reductive evolution of mollicutes. PLoS Genet. 10:e1004363. 10.1371/journal.pgen.1004363 24809820 PMC4014445

[B25] Hahn T. W. Willby M. J. Krause D. C. (1998). HMW1 is required for cytadhesin P1 trafficking to the attachment organelle in *Mycoplasma pneumoniae*. J. Bacteriol. 180, 1270–1276. 9495768 10.1128/jb.180.5.1270-1276.1998 PMC107017

[B26] Hansen E. J. Wilson R. M. Baseman J. B. (1979). Two-dimensional gel electrophoretic comparison of proteins from virulent and avirulent strains of *Mycoplasma pneumoniae*. Infect. Immun. 24, 468–475. 457282 10.1128/iai.24.2.468-475.1979 PMC414325

[B27] Hasselbring B. M. Jordan J. L. Krause D. C. (2005). Mutant analysis reveals a specific requirement for protein P30 in *Mycoplasma pneumoniae* gliding motility. J. Bacteriol. 187, 6281–6289. 10.1128/JB.187.18.6281-6289.2005 16159760 PMC1236621

[B28] Hasselbring B. M. Jordan J. L. Krause R. W. Krause D. C. (2006). Terminal organelle development in the cell wall-less bacterium *Mycoplasma pneumoniae*. Proc. Natl. Acad. Sci. U.S.A. 103, 16478–16483. 10.1073/pnas.0608051103 17062751 PMC1637607

[B29] Hasselbring B. M. Krause D. C. (2007a). Cytoskeletal protein P41 is required to anchor the terminal organelle of the wall-less prokaryote *Mycoplasma pneumoniae*. Mol. Microbiol. 63, 44–53. 10.1111/j.1365-2958.2006.05507.x 17163973

[B30] Hasselbring B. M. Krause D. C. (2007b). Proteins P24 and P41 function in the regulation of terminal-organelle development and gliding motility in *Mycoplasma pneumoniae*. J. Bacteriol. 189, 7442–7449. 10.1128/JB.00867-07 17693502 PMC2168445

[B31] Hasselbring B. M. Sheppard E. S. Krause D. C. (2012). P65 truncation impacts P30 dynamics during *Mycoplasma pneumoniae* gliding. J. Bacteriol. 194, 3000–3007. 10.1128/JB.00091-12 22544269 PMC3370611

[B32] Hatchel J. M. Balish M. F. (2008). Attachment organelle ultrastructure correlates with phylogeny, not gliding motility properties, in *Mycoplasma pneumoniae* relatives. Microbiology 154, 286–295. 10.1099/mic.0.2007/012765-0 18174147

[B33] Hegermann J. Herrmann R. Mayer F. (2002). Cytoskeletal elements in the bacterium *Mycoplasma pneumoniae*. Naturwissenschaften 89, 453–458. 10.1007/s00114-002-0359-2 12384719

[B34] Henderson G. P. Jensen G. J. (2006). Three-dimensional structure of *Mycoplasma pneumoniae*'s attachment organelle and a model for its role in gliding motility. Mol. Microbiol. 60, 376–385. 10.1111/j.1365-2958.2006.05113.x 16573687

[B35] Hu P. C. Cole R. M. Huang Y. S. Graham J. A. Gardner D. E. Collier A. M.. (1982). *Mycoplasma pneumoniae* infection: role of a surface protein in the attachment organelle. Science 216, 313–315. 10.1126/science.6801766 6801766

[B36] Hu P. C. Schaper U. Collier A. M. Clyde W. A. Jr. Horikawa M. Huang Y. S.. (1987). A *Mycoplasma genitalium* protein resembling the *Mycoplasma pneumoniae* attachment protein. Infect. Immun. 55, 1126–1131. 2437033 10.1128/iai.55.5.1126-1131.1987 PMC260479

[B37] Inamine J. M. Loechel S. Hu P. C. (1988). Analysis of the nucleotide sequence of the *P1* operon of *Mycoplasma pneumoniae*. Gene 73, 175–183. 10.1016/0378-1119(88)90323-X 2468577

[B38] Jaffe J. D. Miyata M. Berg H. C. (2004a). Energetics of gliding motility in *Mycoplasma mobile*. J. Bacteriol. 186, 4254–4261. 10.1128/JB.186.13.4254-4261.2004 15205428 PMC421629

[B39] Jaffe J. D. Stange-Thomann N. Smith C. DeCaprio D. Fisher S. Butler J.. (2004b). The complete genome and proteome of *Mycoplasma mobile*. Genome Res. 14, 1447–1461. 10.1101/gr.2674004 15289470 PMC509254

[B40] Jarrell K. F. McBride M. J. (2008). The surprisingly diverse ways that prokaryotes move. Nat. Rev. Microbiol. 6, 466–476. 10.1038/nrmicro1900 18461074

[B41] Jordan J. L. Berry K. M. Balish M. F. Krause D. C. (2001). Stability and subcellular localization of cytadherence-associated protein P65 in *Mycoplasma pneumoniae*. J. Bacteriol. 183, 7387–7391. 10.1128/JB.183.24.7387-7891.2001 11717298 PMC95588

[B42] Jordan J. L. Chang H. Y. Balish M. F. Holt L. S. Bose S. R. Hasselbring B. M.. (2007). Protein P200 is dispensable for *Mycoplasma pneumoniae* hemadsorption but not gliding motility or colonization of differentiated bronchial epithelium. Infect. Immun. 75, 518–522. 10.1128/IAI.01344-06 17043103 PMC1828431

[B43] Kasai T. Hamaguchi T. Miyata M. (2015). Gliding motility of *Mycoplasma mobile* on uniform oligosaccharides. J. Bacteriol. 197, 2952–2957. 10.1128/JB.00335-15 26148712 PMC4542179

[B44] Kasai T. Nakane D. Ishida H. Ando H. Kiso M. Miyata M. (2013). Role of binding in *Mycoplasma mobile* and *Mycoplasma pneumoniae* gliding analyzed through inhibition by synthesized sialylated compounds. J. Bacteriol. 195, 429–435. 10.1128/JB.01141-12 23123913 PMC3554017

[B45] Kawamoto A. Matsuo L. Kato T. Yamamoto H. Namba K. Miyata M. (2016). Periodicity in attachment organelle revealed by electron cryotomography suggests conformational changes in gliding mechanism of *Mycoplasma pneumoniae*. MBio 7, e00243–e00216. 10.1128/mBio.00243-16 27073090 PMC4959525

[B46] Kearns D. B. (2010). A field guide to bacterial swarming motility. Nat. Rev. Microbiol. 8, 634–644. 10.1038/nrmicro2405 20694026 PMC3135019

[B47] Kelley L. A. Sternberg M. J. (2009). Protein structure prediction on the Web: a case study using the Phyre server. Nat. Protoc. 4, 363–371. 10.1038/nprot.2009.2 19247286

[B48] Kenri T. Seto S. Horino A. Sasaki Y. Sasaki T. Miyata M. (2004). Use of fluorescent-protein tagging to determine the subcellular localization of *Mycoplasma pneumoniae* proteins encoded by the cytadherence regulatory locus. J. Bacteriol. 186, 6944–6955. 10.1128/JB.186.20.6944-6955.2004 15466048 PMC522203

[B49] Kenri T. Taniguchi R. Sasaki Y. Okazaki N. Narita M. Izumikawa K.. (1999). Identification of a new variable sequence in the P1 cytadhesin gene of *Mycoplasma pneumoniae*: evidence for the generation of antigenic variation by DNA recombination between repetitive sequences. Infect. Immun. 67, 4557–4562. 10456900 10.1128/iai.67.9.4557-4562.1999 PMC96778

[B50] Kinosita Y. Nakane D. Sugawa M. Masaike T. Mizutani K. Miyata M.. (2014). Unitary step of gliding machinery in *Mycoplasma mobile*. Proc. Natl. Acad. Sci. U.S.A. 111, 8601–8606. 10.1073/pnas.1310355111 24912194 PMC4060671

[B51] Kirchhoff H. Boldt U. Rosengarten R. Klein-Struckmeier A. (1987). Chemotactic response of a gliding mycoplasma. Curr. Microbiol. 15, 57–60. 10.1007/BF01577215

[B52] Koebnik R. (1995). Proposal for a peptidoglycan-associating alpha-helical motif in the C-terminal regions of some bacterial cell-surface proteins. Mol. Microbiol. 16, 1269–1270. 10.1111/j.1365-2958.1995.tb02348.x 8577259

[B53] Krause D. C. Balish M. F. (2004). Cellular engineering in a minimal microbe: structure and assembly of the terminal organelle of *Mycoplasma pneumoniae*. Mol. Microbiol. 51, 917–924. 10.1046/j.1365-2958.2003.03899.x 14763969

[B54] Krause D. C. Leith D. K. Wilson R. M. Baseman J. B. (1982). Identification of *Mycoplasma pneumoniae* proteins associated with hemadsorption and virulence. Infect. Immun. 35, 809–817. 6802761 10.1128/iai.35.3.809-817.1982 PMC351120

[B55] Krause D. C. Proft T. Hedreyda C. T. Hilbert H. Plagens H. Herrmann R. (1997). Transposon mutagenesis reinforces the correlation between *Mycoplasma pneumoniae* cytoskeletal protein HMW2 and cytadherence. J. Bacteriol. 179, 2668–2677. 9098066 10.1128/jb.179.8.2668-2677.1997 PMC179017

[B56] Krunkosky T. M. Jordan J. L. Chambers E. Krause D. C. (2007). *Mycoplasma pneumoniae* host-pathogen studies in an air-liquid culture of differentiated human airway epithelial cells. Microb. Pathog. 42, 98–103. 10.1016/j.micpath.2006.11.003 17261358

[B57] Ku C. Lo W. S. Kuo C. H. (2014). Molecular evolution of the actin-like MreB protein gene family in wall-less bacteria. Biochem. Biophys. Res. Commun. 446, 927–932. 10.1016/j.bbrc.2014.03.039 24650664

[B58] Kürner J. Frangakis A. S. Baumeister W. (2005). Cryo-electron tomography reveals the cytoskeletal structure of *Spiroplasma melliferum*. Science 307, 436–438. 10.1126/science.1104031 15662018

[B59] Layh-Schmitt G. Herrmann R. (1992). Localization and biochemical characterization of the ORF6 gene product of the *Mycoplasma pneumoniae* P1 operon. Infect. Immun. 60, 2906–2913. 1612757 10.1128/iai.60.7.2906-2913.1992 PMC257253

[B60] Layh-Schmitt G. Herrmann R. (1994). Spatial arrangement of gene products of the P1 operon in the membrane of *Mycoplasma pneumoniae*. Infect. Immun. 62, 974–979. 8112872 10.1128/iai.62.3.974-979.1994 PMC186212

[B61] Layh-Schmitt G. Podtelejnikov A. Mann M. (2000). Proteins complexed to the P1 adhesin of *Mycoplasma pneumoniae*. Microbiology 146, 741–747. 10.1099/00221287-146-3-741 10746778

[B62] Letunic I. Doerks T. Bork P. (2015). SMART: recent updates, new developments and status in 2015. Nucleic Acids Res. 43, D257–D260. 10.1093/nar/gku949 25300481 PMC4384020

[B63] Lo W. S. Chen L. L. Chung W. C. Gasparich G. E. Kuo C. H. (2013). Comparative genome analysis of *Spiroplasma melliferum* IPMB4A, a honeybee-associated bacterium. BMC Genomics 14:22. 10.1186/1471-2164-14-22 23324436 PMC3563533

[B64] Manchee R. J. Taylor-Robinson D. (1969). Utilization of neuraminic acid receptors by mycoplasmas. J. Bacteriol. 98, 914–919. 5788718 10.1128/jb.98.3.914-919.1969 PMC315273

[B65] Mayer F. (2006). Cytoskeletal elements in bacteria *Mycoplasma pneumoniae, Thermoanaerobacterium* sp., and *Escherichia coli* as revealed by electron microscopy. J. Mol. Microbiol. Biotechnol. 11, 228–243. 10.1159/000094057 16983198

[B66] Meng K. E. Pfister R. M. (1980). Intracellular structures of *Mycoplasma pneumoniae* revealed after membrane removal. J. Bacteriol. 144, 390–399. 6774963 10.1128/jb.144.1.390-399.1980 PMC294663

[B67] Miyata M. (2008). Centipede and inchworm models to explain *Mycoplasma* gliding. Trends Microbiol. 16, 6–12. 10.1016/j.tim.2007.11.002 18083032

[B68] Miyata M. (2010). Unique centipede mechanism of *Mycoplasma* gliding. Annu. Rev. Microbiol. 64, 519–537. 10.1146/annurev.micro.112408.134116 20533876

[B69] Miyata M. Hamaguchi T. (2016). Prospects for the gliding mechanism of *Mycoplasma mobile*. Curr. Opin. Microbiol. 29, 15–21. 10.1016/j.mib.2015.08.010 26500189

[B70] Miyata M. Nakane D. (2013). Gliding mechanism of *Mycoplasma pneumoniae* subgroup implication from *Mycoplasma mobile*, in Molecular and Cell Biology of Mollicutes, eds Browning G. Citti C. (Norfolk: Horizon Press), 237–252.

[B71] Nagai R. Miyata M. (2006). Gliding motility of *Mycoplasma mobile* can occur by repeated binding to *N*-acetylneuraminyllactose (sialyllactose) fixed on solid surfaces. J. Bacteriol. 188, 6469–6475. 10.1128/JB.00754-06 16952936 PMC1595466

[B72] Nakane D. Adan-Kubo J. Kenri T. Miyata M. (2011). Isolation and characterization of P1 adhesin, a leg protein of the gliding bacterium *Mycoplasma pneumoniae*. J. Bacteriol. 193, 715–722. 10.1128/JB.00796-10 21097617 PMC3021217

[B73] Nakane D. Kenri T. Matsuo L. Miyata M. (2015). Systematic structural analyses of attachment organelle in *Mycoplasma pneumoniae*. PLoS Pathog. 11:e1005299. 10.1371/journal.ppat.1005299 26633540 PMC4669176

[B74] Nakane D. Miyata M. (2009). Cytoskeletal asymmetrical-dumbbell structure of a gliding mycoplasma, *Mycoplasma gallisepticum*, revealed by negative-staining electron microscopy. J. Bacteriol. 191, 3256–3264. 10.1128/JB.01823-08 19286806 PMC2687163

[B75] Ozyamak E. Kollman J. M. Komeili A. (2013). Bacterial actins and their diversity. Biochemistry 52, 6928–6939. 10.1021/bi4010792 24015924 PMC4318550

[B76] Page C. A. Krause D. C. (2013). Protein kinase/phosphatase function correlates with gliding motility in *Mycoplasma pneumoniae*. J. Bacteriol. 195, 1750–1757. 10.1128/JB.02277-12 23396910 PMC3624554

[B77] Pich O. Q. Burgos R. Ferrer-Navarro M. Querol E. Pinol J. (2006). *Mycoplasma genitalium mg200* and *mg386* genes are involved in gliding motility but not in cytadherence. Mol. Microbiol. 60, 1509–1519. 10.1111/j.1365-2958.2006.05187.x 16796684

[B78] Popham P. L. Hahn T. W. Krebes K. A. Krause D. C. (1997). Loss of HMW1 and HMW3 in noncytadhering mutants of *Mycoplasma pneumoniae* occurs post-translationally. Proc. Natl. Acad. Sci. U.S.A. 94, 13979–13984. 10.1073/pnas.94.25.13979 9391138 PMC28418

[B79] Prince O. A. Krunkosky T. M. Krause D. C. (2014). *In vitro* spatial and temporal analysis of *Mycoplasma pneumoniae* colonization of human airway epithelium. Infect. Immun. 82, 579–586. 10.1128/IAI.01036-13 24478073 PMC3911394

[B80] Radestock U. Bredt W. (1977). Motility of *Mycoplasma pneumoniae*. J. Bacteriol. 129, 1495–1501. 14925 10.1128/jb.129.3.1495-1501.1977 PMC235127

[B81] Razin S. Hayflick L. (2010). Highlights of mycoplasma research–an historical perspective. Biologicals 38, 183–190. 10.1016/j.biologicals.2009.11.008 20149687

[B82] Razin S. Jacobs E. (1992). Mycoplasma adhesion. J. Gen. Microbiol. 138, 407–422. 10.1099/00221287-138-3-407 1593256

[B83] Razin S. Yogev D. Naot Y. (1998). Molecular biology and pathogenicity of mycoplasmas. Microbiol. Mol. Biol. Rev. 62, 1094–1156. 9841667 10.1128/mmbr.62.4.1094-1156.1998 PMC98941

[B84] Relich R. F. Balish M. F. (2011). Insights into the function of *Mycoplasma pneumoniae* protein P30 from orthologous gene replacement. Microbiology 157, 2862–2870. 10.1099/mic.0.052464-0 21778204 PMC3353390

[B85] Relich R. F. Friedberg A. J. Balish M. F. (2009). Novel cellular organization in a gliding mycoplasma, *Mycoplasma insons*. J. Bacteriol. 191, 5312–5314. 10.1128/JB.00474-09 19525350 PMC2725587

[B86] Roberts D. D. Olson L. D. Barile M. F. Ginsburg V. Krivan H. C. (1989). Sialic acid-dependent adhesion of *Mycoplasma pneumoniae* to purified glycoproteins. J. Biol. Chem. 264, 9289–9293. 2470754

[B87] Schmidl S. R. Gronau K. Hames C. Busse J. Becher D. Hecker M.. (2010). The stability of cytadherence proteins in *Mycoplasma pneumoniae* requires activity of the protein kinase PrkC. Infect. Immun. 78, 184–192. 10.1128/IAI.00958-09 19858294 PMC2798226

[B88] Seto S. Kenri T. Tomiyama T. Miyata M. (2005). Involvement of P1 adhesin in gliding motility of *Mycoplasma pneumoniae* as revealed by the inhibitory effects of antibody under optimized gliding conditions. J. Bacteriol. 187, 1875–1877. 10.1128/JB.187.5.1875-1877.2005 15716461 PMC1064011

[B89] Seto S. Layh-Schmitt G. Kenri T. Miyata M. (2001). Visualization of the attachment organelle and cytadherence proteins of *Mycoplasma pneumoniae* by immunofluorescence microscopy. J. Bacteriol. 183, 1621–1630. 10.1128/JB.183.5.1621-1630.2001 11160093 PMC95047

[B90] Seto S. Miyata M. (2003). The attachment organelle formation represented by localization of cytadherence protein and formation of electron-dense core in the wild-type and mutant strains of *Mycoplasma pneumoniae*. J. Bacteriol. 185, 1082–1091. 10.1128/JB.185.3.1082-1091.2003 12533484 PMC142798

[B91] Seybert A. Herrmann R. Frangakis A. S. (2006). Structural analysis of *Mycoplasma pneumoniae* by cryo-electron tomography. J. Struct. Biol. 156, 342–354. 10.1016/j.jsb.2006.04.010 16875842

[B92] Shaevitz J. W. Lee J. Y. Fletcher D. A. (2005). *Spiroplasma* swim by a processive change in body helicity. Cell 122, 941–945. 10.1016/j.cell.2005.07.004 16179261

[B93] Shimizu T. Miyata M. (2002). Electron microscopic studies of three gliding mycoplasmas, *Mycoplasma mobile, M. pneumoniae*, and *M. gallisepticum*, by using the freeze-substitution technique. Curr. Microbiol. 44, 431–434. 10.1007/s00284-001-0014-8 12000994

[B94] Sobeslavsky O. Prescott B. Chanock R. M. (1968). Adsorption of *Mycoplasma pneumoniae* to neuraminic acid receptors of various cells and possible role in virulence. J. Bacteriol. 96, 695–705. 4183967 10.1128/jb.96.3.695-705.1968 PMC252361

[B95] Spuesens E. B. Oduber M. Hoogenboezem T. Sluijter M. Hartwig N. G. van Rossum A. M.. (2009). Sequence variations in RepMP2/3 and RepMP4 elements reveal intragenomic homologous DNA recombination events in *Mycoplasma pneumoniae*. Microbiology 155, 2182–2196. 10.1099/mic.0.028506-0 19389769

[B96] Stevens M. K. Krause D. C. (1992). *Mycoplasma pneumoniae* cytadherence phase-variable protein HMW3 is a component of the attachment organelle. J. Bacteriol. 174, 4265–4274. 1624421 10.1128/jb.174.13.4265-4274.1992 PMC206209

[B97] Trachtenberg S. Dorward L. M. Speransky V. V. Jaffe H. Andrews S. B. Leapman R. D. (2008). Structure of the cytoskeleton of *Spiroplasma melliferum* BC3 and its interactions with the cell membrane. J. Mol. Biol. 378, 778–789. 10.1016/j.jmb.2008.02.020 18400234

[B98] Typas A. Sourjik V. (2015). Bacterial protein networks: properties and functions. Nat. Rev. Microbiol. 13, 559–572. 10.1038/nrmicro3508 26256789

[B99] Uenoyama A. Miyata M. (2005). Gliding ghosts of *Mycoplasma mobile*. Proc. Natl. Acad. Sci. U.S.A. 102, 12754–12758. 10.1073/pnas.0506114102 16126895 PMC1192825

[B100] Wada H. Netz R. R. (2009). Hydrodynamics of helical-shaped bacterial motility. Phys. Rev. E Stat. Nonlin. Soft Matter Phys. 80:021921. 10.1103/PhysRevE.80.021921 19792165

[B101] Waldo R. H. III. Popham P. L. Romero-Arroyo C. E. Mothershed E. A. Lee K. K. Krause D. C. (1999). Transcriptional analysis of the *hmw* gene cluster of *Mycoplasma pneumoniae*. J. Bacteriol. 181, 4978–4985. 10438770 10.1128/jb.181.16.4978-4985.1999 PMC93987

[B102] Weisburg W. G. Tully J. G. Rose D. L. Petzel J. P. Oyaizu H. Yang D.. (1989). A phylogenetic analysis of the mycoplasmas: basis for their classification. J. Bacteriol. 171, 6455–6467. 2592342 10.1128/jb.171.12.6455-6467.1989 PMC210534

[B103] Wilson M. H. Collier A. M. (1976). Ultrastructural study of *Mycoplasma pneumoniae* in organ culture. J. Bacteriol. 125, 332–339. 54354 10.1128/jb.125.1.332-339.1976 PMC233367

[B104] Zhao X. Norris S. J. Liu J. (2014). Molecular architecture of the bacterial flagellar motor in cells. Biochemistry 53, 4323–4333. 10.1021/bi500059y 24697492 PMC4221660

